# Embryonic development and inviability phenotype of chicken-Japanese quail F_1_ hybrids

**DOI:** 10.1038/srep26369

**Published:** 2016-05-20

**Authors:** Satoshi Ishishita, Keiji Kinoshita, Mikiharu Nakano, Yoichi Matsuda

**Affiliations:** 1 Avian Bioscience Research Center, Graduate School of Bioagricultural Sciences, Graduate School of Bioagricultural Sciences, Nagoya University, Furo-cho, Chikusa-ku, Nagoya, Aichi, 464-8601, Japan; 2 Laboratory of Animal Genetics, Department of Applied Molecular Biosciences, Graduate School of Bioagricultural Sciences, Nagoya University, Furo-cho, Chikusa-ku, Nagoya, Aichi, 464-8601, Japan

## Abstract

Interspecific hybrid incompatibility, including inviability and sterility, is important in speciation; however, its genetic basis remains largely unknown in vertebrates. Crosses between male chickens and female Japanese quails using artificial insemination can generate intergeneric hybrids; however, the hatching rate is low, and hatched hybrids are only sterile males. Hybrid development is arrested frequently during the early embryonic stages, and the sex ratio of living embryos is male-biased. However, the development and sex ratio of hybrid embryos have not been comprehensively analyzed. In the present study, we observed delayed embryonic development of chicken-quail hybrids during the early stage, compared with that of chickens and quails. The survival rate of hybrids decreased markedly during the blastoderm-to-pre-circulation stage and then decreased gradually through the subsequent stages. Hybrid females were observed at more than 10 d of incubation; however, the sex ratio of hybrids became male-biased from 10 d of incubation. Severely malformed embryos were observed frequently in hybrids. These results suggest that developmental arrest occurs at various stages in hybrid embryos, including a sexually non-biased arrest during the early stage and a female-biased arrest during the late stage. We discuss the genetic basis for hybrid inviability and its sex bias.

Mating of genetically differentiated individuals sometimes contributes to the diversification and adaptability of species; however, it often causes abnormalities, such as lethality, sterility, and malformation, collectively termed hybrid incompatibility^1^,^2^. Hybrid lethality and/or sterility prevent gene flow between different species. Furthermore, reduction of fertility and viability, and developmental failure lower the hybrid fitness; these adverse effects of hybridization may reinforce reproductive isolation by promoting the evolution of assortative mating in sympatric species^1^,^2^,^3^. Therefore, hybrid incompatibility is important for reproductive isolation, leading to speciation.

Previous genetic studies on hybrid dysgenesis revealed several molecular events associated with abnormal growth and development in hybrids, such as transposon derepression during embryogenesis, and abnormal genomic imprinting^4^,^5^. Failure of transposon silencing is possibly involved in hybrid incompatibility in a wide range of biological taxa^5^, suggesting that common molecular mechanisms underlie hybrid incompatibility. Furthermore, abnormal phenotypes of hybrids usually become more severe in hybrids with heterogametic sex than in those with homogametic sex in *Drosophila*, Lepidoptera, birds, and mammals, which is known as Haldane’s rule^6^,^7^,^8^,^9^,^10^,^11^. Studies on hybrid inviability using model organisms have provided important insights into the genetic basis of Haldane’s rule; however, in birds, the genetic basis of Haldane’s rule remains unclear, although there are many reports on avian hybrids^8^,^12^,^13^,^14^,^15^.

Interspecific and intergeneric hybridizations have been performed in a variety of bird families^14^. Hybrid inviability and sterility in birds is more severe in the ZW heterogametic sex than ZZ homogametic sex^7^,^8^,^14^. Furthermore, hybrid inviability in birds occurs frequently during the embryonic stage^16^,^17^,^18^,^19^,^20^,^21^. Despite many reports on hybrid inviability and sterility, embryonic development of bird hybrids has not been studied in detail.

The female heterogametic ZW sex determination system in birds differs from the male heterogametic XY sex determination system in mammals^10^,^11^. In addition, it is likely that genomic imprinting is absent in birds, unlike in mammals^22^,^23^,^24^. Thus, bird hybrids would provide new genetic insight into hybrid incompatibility.

The present work focuses on the embryonic development of interspecific hybrids from crosses between chickens (*Gallus gallus domesticus*) and Japanese quails (*Coturnix japonica*), hereafter referred to as quails. Both these species belong to the family Phasianidae, and were estimated, using molecular phylogenetic analysis, to have diverged 35 million years ago (MYA)^25^. Adult male and female chickens usually weigh approximately 1.5–3.5 kg^26^. Adult male and female quails weigh 100–130 g and 120–160 g, respectively^27^. Incubation periods are 21 d in chickens and 17 d in quails^28^, and periods of maturity are 18–25 wk in chickens and 6–7 wk in quails^27^,^29^. The number of chromosomes is 78 (2 n = 78) in both chickens and quails^30^. Molecular cytogenetic analysis showed that the chromosome structures are highly conserved between chickens and quails; however, large inversions are present in several chromosomes between the two species^30^,^31^. Chickens and quails never hybridize in the wild; however, F_1_ hybrids between male chickens and female quails can be generated by artificial insemination^32^,^33^. To our knowledge, hybridization by reverse crosses (female chickens × male quails) has never been successful. Hybrid inviability evolves much slower in birds than in mammals^34^, and complete inviability occurs in the bird hybrid between species diverged 11–55 MYA^14^.

The known features of chicken-quail F_1_ hybrids are as follows. The fertilization rate in the interspecific crosses is low, ranging from 5.4 to 25.3%^33^,^34^,^35^,^36^,^37^,^38^. The fertilization rate is largely different among breeds of chickens and quail strains used for hybridization^33^,^37^,^39^. In most fertilized eggs, embryonic development is arrested around the formation of the extraembryonic membrane and/or blood islands, and in a small fraction of the remaining fertilized eggs, hybrids are hatched^37^. Other studies also showed the low hatching rate and 19-d incubation period of the hybrid^33^,^36^,^38^. Although both hybrid male and female embryos are observed during the early stage, hatched hybrids are only males^33^,^37^,^40^. This sex bias in the viability agrees with Haldane’s rule. The full body weight of hatched hybrids is approximately 350–480 g at 120–150 d of age^33^,^37^. Hybrid males exhibit sterility with testes that are much smaller than those of the parental species^41^. Nondisjunction has not been observed in either chicken or quail chromosomal sets^40^,^42^.

The genetic causes of the incompatibility in chicken-quail hybrids may be common with those in hybrids between other bird species. Therefore, this hybrid is a good animal model for studying hybrid inviability in birds. Furthermore, chicken-quail hybrids would be an animal model to understand genetic basis underlying the difference in rates of evolution of hybrid inviability between birds and mammals: interspecific hybrid inviability evolves much faster in mammals than in birds^34^. However, no comprehensive analysis of the embryonic development of chicken-quail hybrids has been conducted to date. Thus, in the present study, we observed chicken-quail F_1_ hybrid embryos at various incubation times and examined sex bias in the frequency of embryonic lethality and anomalies of the hybrid.

## Results

### Fertilization and survival rates at various incubation times

We initially examined the fertilization rate in interspecific crosses, and analyzed the developmental status of hybrid embryos at various incubation times. First, eggs were opened at 3–7 d of incubation, and the developmental status of hybrids at 3 d of incubation was estimated. Of the 2,344 incubated eggs, 31.2% eggs were fertilized (Table S1). In most of the fertilized eggs, embryos stopped developing before the formation of discernible embryonic structures: 43.4% of the fertilized eggs were arrested during the extraembryonic membrane formation stage, and 35.2% during the extraembryonic membrane and blood island formation stages (Table S1). The frequencies of dead and living embryos at 3 d of incubation were estimated to be 5.7 and 15.6% of the fertilized eggs, respectively (Table S1). Previous studies on chicken-quail F_1_ hybrids showed that the fertilization rate in this interspecific cross was 5.4–25.3%^32^,^33^,^37^,^38^ and that development was arrested in 42.9–62.8% of fertilized eggs during the extraembryonic membrane formation stage and 19.0–29.2% of fertilized eggs during the stage of the formation of both extraembryonic membranes and blood islands^37^; and approximately 70% of fertilized eggs died during 0–2 d of incubation^38^. Thus, our data are in agreement with the data of the previous studies, and consistently showed that a high frequency of developmental arrest occurs during early embryonic stages in the hybrid.

Next, we estimated the survival rates of hybrids at different points of time by opening the eggs at 18−20 d of incubation. We noted that chicken-quail F_1_ hybrids usually hatched at 19 d of incubation^33^; however, most embryos died before hatching. Therefore, in this study, the incubation time at which developmental arrest occurred in the hybrid embryos was examined based on their morphology, skin pigmentations, and feather developments. Fertilized eggs accounted for 24.3% of the 1,664 incubated eggs (Table 1). In 74.3% of the fertilized eggs, embryonic development was arrested during the extraembryonic membrane and/or blood-island formation stage (Table 1). In these eggs, embryonic structures were not observed macroscopically, which suggests that the developmental arrest occurred within 0–2 d of incubation. Of the fertilized eggs, 4.9% were arrested during 2–4 d of incubation, 5.7% during 5–9 d, 1.2% during 10–11 d, and 9.6% during 12–18 d, and 4.2% were hatched (Table 1). Accordingly, the survival rates of F_1_ hybrids were estimated to be 25.7% for 2 d, 20.7% for 5 d, 15.1% for 10 d, and 13.8% for 12 d. Of the 70 fertilized control quail eggs, 82.9% hatched (Table 2). The survival rates at 2, 5, 10, and 12 d of incubation were estimated to be 94.3%, 94.3%, 92.9%, and 92.9%, respectively.

**Figure 1:**
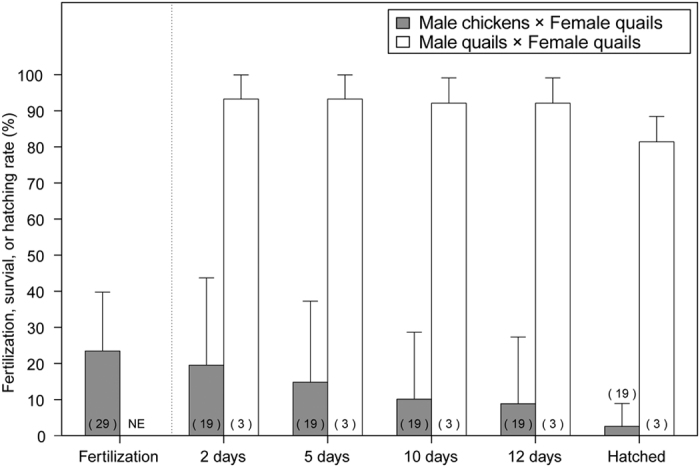
The average rates of fertilization, survival of embryos at various incubation times, and hatching in interspecific hybridization. Columns represent average rates of fertilization, survival of embryos at 2, 5, 10, and 12 d of incubation and hatching. Gray columns, chicken-quail interspecific crosses; white columns, crosses between male and female quails; whiskers, standard deviation. The number within each parenthesis, the number of female quails used for crossing; NE, not examined. Survival and hatching rates were calculated from the fertilized eggs, and not from the incubated eggs. Female quails that laid 34 or more eggs were used for the analysis of the average fertilization rate in interspecific hybridization. Fractions of female quails, which laid 10 or more fertilized eggs were used for the analysis of average survival and hatching rates. Three female quails mated with male quails laid 15–29 fertilized eggs.

Previous studies suggested a large difference in fertilization rate between female quail individuals^37^,^39^. Thus, we analyzed the fertilization, survival, and hatching rates for each quail individually. In the present study, the fertilization rate was 0–64.3% [23.5 ± 16.4% (mean ± standard deviation)] for 29 female quails that laid more than 34 eggs (Figs 1 and S1). Of the 29 female quails, 19 females that laid more than 10 fertilized eggs were used for the following analysis. Survival rates of hybrids were 0–89.2% (19.5 ± 24.3%) at 2 d of incubation, 0–83.8% (14.8 ± 22.6%) at 5 d, 0–75.7% (10.2 ± 18.8%) at 10 d, and 0–75.7% (8.9 ± 18.8%) at 12 d, and the hatching rate was 0–27.0% (2.6 ± 6.6%) (Figs 1 and S2). For control quails, survival rates were 86.7–100% (93.3 ± 6.7%) at 2 d of incubation, 86.7–100% (93.3 ± 6.7%) at 5 d, 86.7–100% (92.1 ± 7.0%) at 10 d, and 86.7–100% (92.1 ± 7.0%) at 12 d, and the hatching rate was 73.3–86.2% (81.3 ± 7.0%) (Fig. 1). Therefore, our data demonstrated that the survival, hatching, and fertilization rates differed greatly among individual female quails.

### Embryonic development of the hybrids

**Figure 2:**
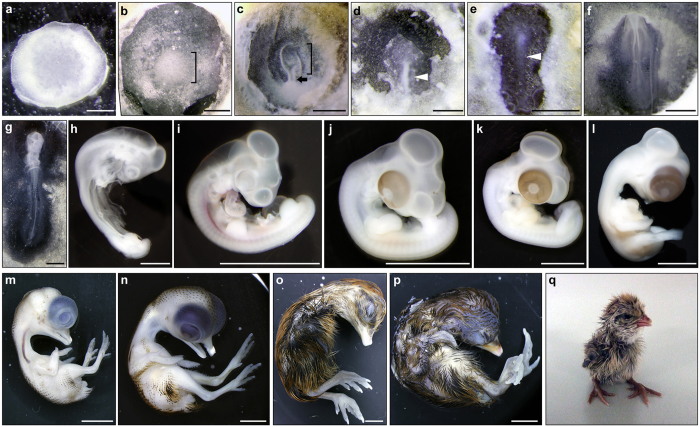
The embryonic development of chicken-quail F_1_ hybrids. (**a–l**) Representative images of hybrid embryos with no apparent morphological abnormalities at stages X (**a**), XIII (**b**), XIV (**c**), 3 (**d**), 4 (**e**), 7 (**f**), 11 (**g**), 17 (**h**), 20 (**i**), 25 (**j**), 27 (**k**), 29 (**l**), which were observed at 0, 8.5, 8, 18, 25, and 27 h, and 2, 3, 4, 5, 6, and 7 d of incubation, respectively. The embryos in (**a–e**) and (**g**) were photographed from the ventral side, and the embryo in (**f**) was from the dorsal side. The hypoblast was observed in the central regions of blastoderms at stages XIII (**b**) and XIV (**c**) (square brackets), and a cellular bridge connecting the hypoblast with the area opaca is formed in the posterior region of the blastoderm at stage XIV (**c**) (arrow). A primitive streak is being elongated at stage 2 (**d**), and fully elongated at stage 4 (**e**) (arrowheads). (**m–p**) Representative images of hybrid embryos at 10 (**m**), 12 (**n**), 14 (**o**), and 16 (**p**) d of incubation. (**q**) Representative images of newborn hybrids. The hybrid was hatched at 19.5 d of incubation. Scale bars, 1 mm in (**a–h**) and 5 mm in (**i–p**).

**Figure 3:**
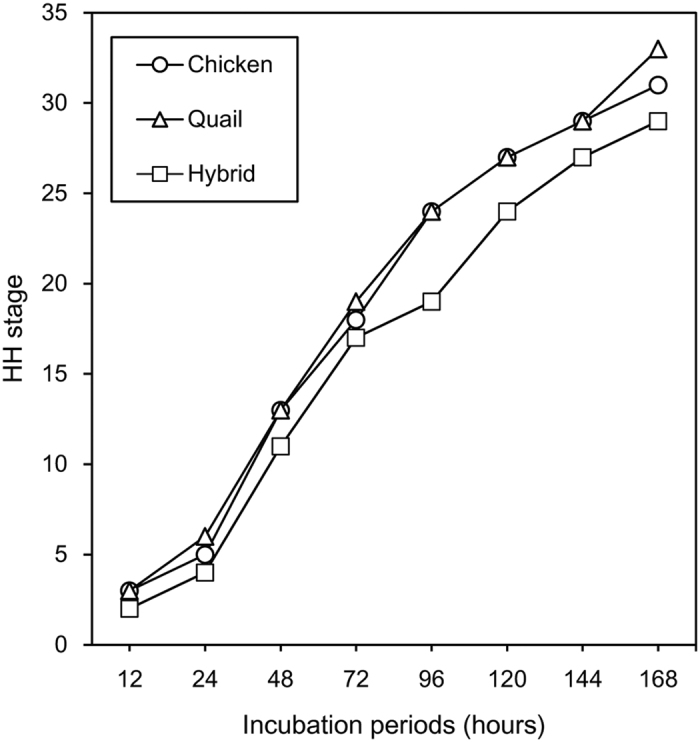
Comparison of the developmental status among chickens, quails, and hybrid embryos. Circles, triangles, and rectangles indicate the stages of chicken, quail, and hybrid embryos at given incubation periods ranging from 12 h to 7 d. Please see Methods and Table S2.

The developmental status was examined at various incubation periods, ranging from 0 d to 19 d, for hybrid embryos that exhibited no apparent morphological anomalies. In this analysis, hybrid embryos at 0–7 d of incubation were staged according to the criteria of chicken staging, as described in Methods. Embryos were not staged after 7 d of incubation because it was difficult to determine the stages precisely by comparing embryo morphology between hybrids and parental species during later developmental stages. At 0 h of incubation, the morphology of hybrid embryos was similar to that of quail blastoderms at EGK stage X (called stage X hereafter) (Figs 2a and S3,S4); however, diameters (3.29 ± 0.23 mm) (mean ± standard deviation) of hybrid blastoderms at stage X were small, compared with those (3.64 ± 0.15 mm) of quail blastoderms at stage X (Mann–Whitney *U*-test, *P* = 0.0284). Compared to parental embryos, the developmental delay was observed in hybrid embryos from 12 h to 7 d of incubation time (Fig. 3, Table S2). In 10–11-d embryos, increased pigmentation was found in feathers and feather buds around the rear sides of the neck, trunk and tail, whereas feathers were still short (Fig. 2m). The development at 10–11-d hybrid embryos was almost equivalent to that of 8–9-d quail embryos in terms of pigmentation and feather development^43^. In 12-d hybrid embryos, skin pigments spread to lateral sides of the trunk, leg, and wing, and feathers were more elongated (Fig. 2n), and in 14- and 16-d hybrid embryos, elongated feathers coated the whole area of the skin (Fig. 2o,p), and hybrids were hatched during 18–21 d of incubation (Fig. 2q, Table S2); however hybrid chicks hatched at 21 d died soon after birth. Because quails were hatched mainly at 17–18 d and chickens at 21 d of incubation (Table S2), the incubation period in hybrids is shorter than that in chickens and is longer than that in quails, as reported previously.

### Sex ratio in hybrids and sex differences in the body weight of hybrids

**Figure 4:**
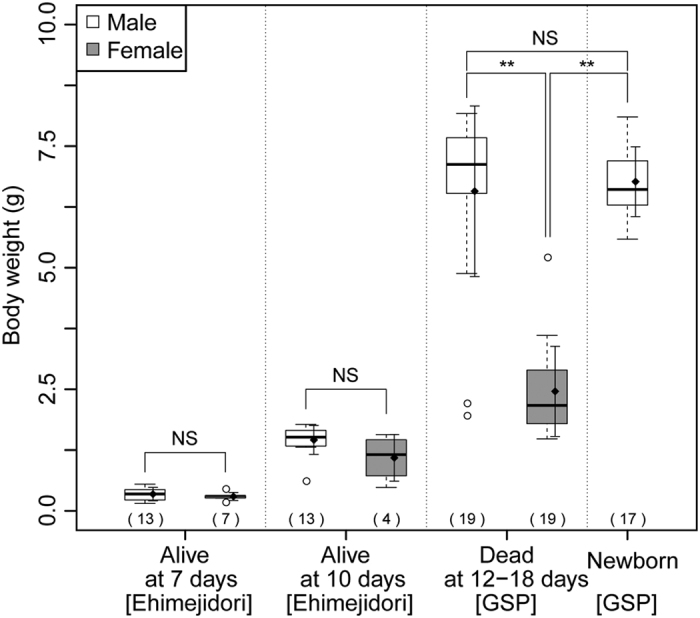
Comparison of the body weight between male and female hybrid embryos. The boxes represent the body weight of hybrid males (white) and females (gray), which were alive at 7 and 10 d, and dead at 12–18 d, and the body weight of newborn hybrid males. The paternal chicken breeds/lines are shown in square brackets. The central bar of each box indicates the median, the lower and upper margins of each box are the first (Q1) and third (Q3) quartile, respectively, the whiskers with dotted lines represent Q1 - 1.5 × interquartile range (IQR) and Q3 + 1.5 × IQR, and external points are outliers. The number of embryos in each boxplot is indicated in parenthesis. Rectangles and whiskers with solid lines represent mean values and ± 1 standard deviation. Double asterisks indicate the significant difference in the body weight with *P* < 0.01; NS, no significant difference in the body weight (*P* > 0.05).

Temporal changes in the sex ratio of hybrids were examined in the samples used for the analysis of survivability of hybrid embryos at various incubation periods. The sex ratio was not biased at 2 d (Pearson’s chi-squared test, *P* = 0.596) and 5 d (*P* = 0.513) of incubation, but it was male-biased at 10 d (*P* = 0.030) and 12 d (*P* = 0.033) of incubation (Table 3). All the hatched hybrids were males. The sex ratios of living embryos at 0 h and 2–3, 5, and 7 d of incubation were analyzed by opening eggs at these incubation periods. The sex ratio was unbiased at 0 h (*P* = 0.354), 2–3 d (*P* = 0.336), 5 d (*P* = 0.433), and 7 d (*P* = 0.274) of incubation (Table S3). To compare the growth of hybrid males and females, their weights were measured. The weights of 7-d living embryos did not differ between males (0.34 ± 0.14 g) (mean ± standard deviation) and females (0.30 ± 0.08 g) (Mann–Whitney *U*-test, *P* = 0.606), and in a group of living hybrids at 10 d of incubation, no significant differences were found in weight between male (1.46 ± 0.30 g) and female (1.09 ± 0.48 g) embryos (*P* = 0.113) (Fig. 4). In dead hybrid embryos at 12–18 d of incubation, development was arrested in most male embryos after full or partial yolk sac absorption; by contrast, development was arrested in most female embryos before the beginning of yolk sac absorption. Weights differed significantly between male (6.57 ± 1.75 g) and female (2.46 ± 0.93 g) embryos (*P* = 3.22 × 10^−6^, nominal significance level of 0.01 is 3.3 × 10^−3^) (Fig. 4). Weights were similar between dead male embryos and newborn chicks (6.77 ± 0.72 g) (*P* = 0.318, nominal significance level of 0.1 is 0.067), but significantly lower in female dead embryos than in newborn chicks (*P* = 3.10 × 10^−7^, nominal significance level of 0.01 is 0.0067) (Fig. 4).

We observed 2 living hybrid females at 12 and 16 d of incubation, which weighed 1.52 g and 2.14 g, respectively. Furthermore, we obtained 2 living hybrid males (BW 2.22 g and 2.65 g) at 12 d of incubation and one 14-d (3.65 g) and one 16-d (3.75 g) male.

### Developmental arrest during the blastoderm-to-pre-circulation stage in the hybrid

**Figure 5:**
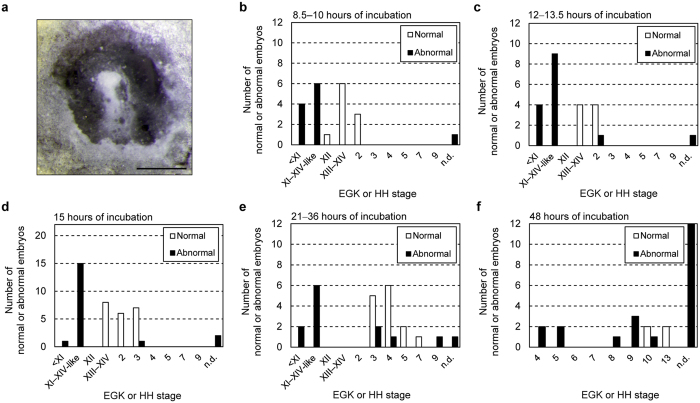
Developmental arrest of hybrids during early embryogenesis. (**a**) Representative image of an abnormal stage XI–XIV-like blastoderm at 21 h of incubation. Scale bar, 1 mm. (**b**–**e)** The number of blastoderms with normal morphology (white columns) and those with abnormal morphology (black columns) at 8.5–10 h (**b**), 12–13.5 h (**c**), 15 h (**d**), 21–36 h (**e**), and 48 h (**f**) of incubation. Embryonic stages are indicated below columns. <XI, stage X or earlier; XI–XIV-like, stages equivalent to stages XI–XIV; n.d., not determined.

Developmental arrest was frequently observed in the early stages of hybrid development, including the blastoderm, somite, and pre-circulation stages (Figs S4 and S5). In a fraction of hybrid embryos incubated from several hours to 2 days, developmental arrest occurred at stage X or earlier (stages VI–IX) (Fig. S4). In addition, we observed many abnormal blastoderms that were similar to stage-XI–XIV blastoderms (Figs 5a and S4), during which the pre-primitive streak and hypoblast are formed. In the stage-XI–XIV-like blastoderms of hybrids, a hypoblast-like cell layer was formed; however, the primitive streak was not formed. Furthermore, epithelia were often swollen or protruded around the center of the ventral (yellow yolk) side of these blastoderms. It was difficult to determine the stages of these blastoderms accurately owing to their abnormal morphologies. Blastoderms with normal morphology accounted for 47.6% of the hybrid embryos at 8.5–10 h of incubation, 34.8% at 12–13.5 h, 52.5% at 15 h, 51.9% at 21–36 h, and 16.0% at 48 h (Fig. 5b–f). Stage XI–XIV-like blastoderms accounted for 54.5% of the abnormal embryos at 8.5–10 h of incubation, 60.0% at 12–13.5 h, and 78.9% at 15 h (Fig. 5b–d). At 21–36 h of incubation, hybrid embryos with normal morphology reached stage 3 or later (stages 4, 5, and 7); however, a substantial fraction of abnormal hybrid embryos did not reach stage 3. Blastoderms at stage X or earlier, and those at stage XI–XIV-like stages, accounted for 15.4% and 46.2% of the morphologically abnormal embryos, respectively, and the remaining abnormal embryos reached stage 3 or later (15.4% at stage 3, 7.7% at stage 4, and 7.7% in stage 9) (Fig. 5e). At 48 h of incubation, hybrid embryos with normal morphology reached stages 10–13, and 42.9% of abnormal hybrid embryos were arrested at stages 4–10; however, the stages of the remaining abnormal embryos could not be determined because of early developmental arrest and their subsequent degeneration (Fig. 5f).

At 15 h of incubation, the number of male and female blastoderms, and those of unknown sex were 14, 25, and 1, respectively. There were six abnormal male blastoderms and 13 abnormal female blastoderms, accounting for 42.9% and 52.1% of the total, respectively, with no significant difference between the sexes (Fisher’s exact test, φ = 0.088, *P* = 0.741).

### High frequency of malformation and the sex ratio of malformed hybrids

Various types of malformations were observed, such as general malformation, head malformation, eventration, abnormal limbs and eyes, and beak malocclusion in hybrid embryos incubated for 2–4 d or more (Fig. S6). During the analysis of embryo survivability at various incubation periods shown in Table 1, 2, malformation was observed in 26 (25.0%) of 104 hybrid embryos, containing 3 malformed chicks, but in none of 66 quail embryos. The frequencies of malformation were 21.3% for male and 38.1% for female embryos (Table S4). Frequencies of malformation did not differ between the sexes for all embryos (Fisher’s exact test, φ = 0.185, *P* = 0.104) and for embryos at more than 10 d of incubation (φ = 0.142, *P* = 0.373) (Table S4); however, further analyses using a larger number of samples are necessary to conclude whether the sex ratio of malformed hybrids is unbiased.

## Discussion

We have shown the low fertilization rate in interspecific crosses between chickens and quails and the low survival rate of hybrid embryos at the early stage, as shown in previous studies^33^,^36^,^37^,^38^. There were large individual differences between female quails in the fertilization rate, survival rate of hybrids at various points of incubation time, and hatching rate of hybrids. The GSP chicken line, which was used for this analysis, has been maintained as a closed colony and is highly inbred^44^,^45^; however, quails used in the analysis were a commercial line exhibiting high genetic heterogeneity^46^,^47^. Therefore, the individual differences found in this study may be attributed mainly to the difference in their genetic background; however, we cannot exclude the possibility that non-genetic individual differences in female quails, such as physiological conditions, cause these individual differences.

The present study firstly showed the delay in development of hybrid embryos during 12 h to 7 d, compared with the parental species. In addition, blastoderms at 0 h of incubation were slightly smaller in hybrids than in quails, suggesting that hybrid development was already delayed at the time of spawning. We speculate that the developmental delay was caused by inappropriate interactions of development-related genes from chickens with those from quails.

The sex ratio in living hybrid embryos was not biased until at least 7 d of incubation, and became biased from 10 d of incubation. A sex-based difference was observed for the weight of dead 12–18 d-old hybrid embryos: the weight was much lower in females than males. These results collectively suggest that a female-biased embryonic lethality begins from 7–10 d of incubation, and the growth of surviving hybrid females stops or is suppressed after an incubation period of more than 10 days, resulting in small dead embryos. Previous studies showed that the male-bias in sex ratio is observed from 3 d of incubation: males accounted for 74.1% of the 27 hybrid embryos^37^. We speculate that the difference between the results of previous studies and the present study results from the difference of genetic background in chicken and quail lines used for mating. Detailed analyses of factors causing female-specific embryonic lethality should be conducted in future studies.

We observed a high frequency of developmental arrest during the early stage in hybrid embryos: 75–80% of hybrid embryos were arrested by 2 d of incubation, at which point normally developed hybrid embryos reached stages 9–13. We observed abnormal hybrid embryos during the primitive streak to pre-circulation stages (stages 4–10) in a fraction of hybrid embryos at 2 d of incubation. Abnormal blastoderms at stage X or earlier, and those at stages XI–XIV were observed in a fraction of hybrid embryos at 21–36 h of incubation, in addition to abnormal embryos during the primitive streak to somite stages. These results suggest that developmental arrest occurs at various stages in early embryonic development. In addition, it is possible that hybrid embryos were arrested during the cleavage stages in the eggs, which were regarded as being unfertilized. Stages XI–XIV represent the pre-primitive streak stages, and during these and subsequent primitive streak stages, cells undergo dynamic movement, proliferation, and differentiation, resulting in the formation of the second body axis and three germ layers^48^,^49^,^50^. We suggest that the abnormal morphologies of stage XI–XIV-like blastoderms would be caused by aberrant movement, proliferation, and/or differentiation of epiblast and/or hypoblast cells that are associated with a defective primitive streak formation. This hypothesis should be validated by further experiments, such as analyses of expression levels and expression sites of genes related to primitive streak formation. In the present study, hybrid embryos were observed by opening eggs at predetermined times, and continual time course observations were not performed. Continual observation of hybrid embryos throughout the early period using an *ex vivo* system may allow us to determine the stage of developmental arrest more accurately.

We have demonstrated a high occurrence of malformations in hybrid embryos. A variety of malformation phenotypes lead us to speculate that various molecular pathways involved in the embryonic development are affected in hybrids. Although severe malformations, such as eventration and head malformation, could cause lethality, it is unclear whether they are the primary causes of embryonic lethality.

Developmental arrest occurred at various stages in hybrid embryos in both sexes, and was female-biased from the late stage. We discuss the genetic and developmental basis of this hybrid inviability. First, as the Dobzhansky–Muller (DM) model proposes^2^,^51^, abnormal interallelic and/or intraallelic interactions may cause hybrid incompatibility in chicken-quail hybrids. A variety of gene-gene interactions may be affected in this hybrid, and the point at which hybrid development is arrested may depend on the genetic background of the parents. It is conceivable that genes encoding proteins with a high percentage of amino-acid substitutions between chickens and quails are involved in abnormal genetic interactions as proposed in the DM model. It has been reported in various species that genes related to immune response and reproduction evolve rapidly^52^. Thus, we speculate that rapidly evolving genes involved in embryonic development, which may have functions in other biological processes, such as immune response and reproduction, are the causative genes of developmental arrest of the hybrid. In addition, like the *Drosophila* P-M system of hybrid dysgenesis^53^, differentiation of transposable elements and their genetic/epigenetic control mechanisms between the species may underlie the early developmental arrest of chicken-quail hybrids. Comparative genomic studies of chickens and quails would allow us to test these possibilities. An alternative possibility is insufficient egg activation after fertilization. Birds usually employ a polyspermy system for fertilization, in which multiple sperms enter the ovule^21^. In this system, only one sperm-derived pronucleus fuses with the oocyte-derived pronucleus (syngamy), and the other sperms contribute to egg activation and subsequent embryonic development^54^. Intracytoplasmic sperm insemination (ICSI) with a single sperm results in early developmental arrest in quails; however, the arrest is overcome by co-injection of a sperm nucleus with a sperm extract^54^. In the fertilized eggs collected from the same female quails, some were arrested during the blastoderm-to-pre-circulation stage and the others passed through these stages. This stochastic phenotype is alternatively explained by the difference in the number of sperms that reach infundibulum in each fertilization event: if a sufficient number of sperm enter into eggs, the eggs are activated and subsequently develop normally; otherwise, embryos are arrested during the early stage. Anti-sperm immunoreaction in the female tract, which may disturb sperm storage in the oviduct, is a proposed cause of failure of fertilization in interspecific hybridization^21^,^55^.

Next, we discuss the genetic basis for the female-biased inviability, which follows Haldane’s rule. At present, there are several representative theories for Haldane’s rule, such as the dominance theory, male-faster theory, and meiotic drive^3^. The dominance theory proposes that recessive incompatibility gene(s) located on the X or Z chromosome have ‘dominance’ effects in hybrids with heterogametic sex (XY in mammals and ZW in birds), which results in preferred inviability or sterility in hybrids with the XY or ZW sex chromosomes^56^. According to this theory, Z-linked recessive incompatibility genes may cause female-biased inviability in chicken-quail hybrids. Recent genome-wide transcriptome analyses of various bird species showed “fast-Z evolution of gene expression” in birds: the Z chromosome-linked genes expression levels have more diversified than those of autosomal genes, and the diversification of Z-linked genes expression levels is remarkable in the heterogametic sex^57^, suggesting that the adjustment of Z-linked gene expression is more difficult in female than male hybrids. Here, the sexual differentiation of gonads occurs during 6 to 10 d in chicken embryos^58^. The sexual differentiation of gonads may influence the sexual differentiation of somatic tissues. Thus, it is conceivable that an inappropriate Z chromosome-linked gene dosage in sexually differentiated somatic cells affects the developmental process in females, which may cause female-biased lethality during the late embryonic stage. Comparison of sexually-biased gene expression between chicken and quail embryos should provide evidence to understand the genetic cause of female-biased developmental arrest in the hybrid.

In summary, the present results show that developmental arrest occurs at various stages in chicken-quail F_1_ hybrid embryos, including a non-sexually biased arrest during the early stage and a female-biased arrest during the late stage. Our findings provide fundamental information for improved understanding of the genetic basis of hybrid inviability and its sexual bias in birds. Future studies will describe hybrid phenotypes at molecular and genetic levels using molecular biological techniques, such as whole-transcriptome analysis by RNA-sequencing and whole-mount *in situ* hybridization with development-related gene probes.

## Methods

### Animals

Animal care and all experimental procedures were approved by the Animal Experiment Committee, Graduate School of Bioagricultural Sciences, Nagoya University (approval numbers: 2009101401, 2010031802, 2011031415, 2012031624, 2013022818, 2014021004, 2015030219), and the experiments were conducted according to Regulations on Animal Experiments at Nagoya University. Thirteen groups (A–M) of female quails were used for artificial insemination (AI) during 16 sampling periods. Purposes of the use of eggs obtained in these sampling periods are indicated in Table S5. Japanese quails were a commercial line purchased from local hatcheries for groups A–E (Kato-farm, Toyohashi, Japan) and F–M (Motoki Corporation, Saitama, Japan), and used at 2-to-12 mo old. To collect chicken semen, adult male chickens of Ehimejidori (Japanese native chicken breed)^59^, the NH-413 line (dystrophic New Hampshire chickens, line 413)^60^, the BL-E line (long-term closed colony of Brown Leghorn breed)^61^, and the GSP line (inbred line of Fayoumi breed)^62^ were used. These chicken breeds and lines are maintained at the Avian Bioscience Research Center, Nagoya University. Chicken breeds/strains used for AI during each sampling period are also listed in Table S5. In addition to the 16 sampling periods, hybrid embryos (female quails of groups H-J × Ehimejidori or BL-E line males) at 7.5-10 h of incubation and hybrid embryos (female quails of group K × GSP line males) at 12–13.5 h were observed for analyzing early embryogenesis of the hybrid. Chickens and quails were maintained with free access to water and a commercially available diet. The photoperiod was set at 14:10 h L:D, and room temperature was controlled at approximately 25 °C. F_1_ hybrids were killed soon after hatching.

### Artificial insemination

AI was performed twice or three times per week. Chicken semen was collected just before AI from 5–15 adult males of each strain, and all samples were pooled. After addition of gentamicin into pooled semen to a final concentration of 10 μg/ml, we injected 50–100 μl semen into the vagina of each quail using a syringe. To avoid the excretion of semen from quail vaginas due to egg laying immediately after injection, AI was conducted during the last 1–2 h of a light period, when most female quails had finished laying eggs on that day.

### Egg preservation and incubation

After identifying the parental females of eggs, laid eggs were stored at 12 °C until incubation or observation. Incubation of eggs was started within 8–10 d of storage, and was carried out at 37.6 °C and 70% relative humidity, with rocking at an angle of 90° at 30-min intervals.

### Fertilization and survival rates at various incubation times

By observing eggs that were opened at 3–7 d of incubation, the fertilization rate and developmental status of hybrids at 3 d of incubation were estimated. Semen from Ehimejidori and NH-413 males were used for AI. It should be noted that hybrid embryos could be observed by macroscopic examination from around 2 d of incubation and that when embryos were dead in the eggs incubated for more than 3 d, we judged whether they were alive at 3 d of incubation from their morphology. By observing the eggs that were opened at 18–20 d of incubation, the fertilization and survival rates of hybrids at different times were estimated. Semen from GSP males were used for AI in this experiment. For controls of this analysis, we incubated eggs from 3 pairs of quails. It was determined by egg candling at 4–5 d of incubation whether early embryogenesis progressed normally, and eggs that were not fertilized or contained dead embryos were opened at that point. The survival rate was a frequency (%) of living embryos in fertilized eggs, and the hatching rate was a frequency (%) of hatched embryos in fertilized eggs.

### Definition of unfertilized eggs and dead embryos

When blastoderms were not observed in the eggs that were not incubated or incubated for less than a dozon hours, and when extraembryonic membranes, blood islands, blood vessels, or embryos were not observed in the eggs after a longer incubation period, these eggs were categorized as unfertilized eggs. Of note, it is possible that fertilized eggs that stopped developing during the cleavage stages were considered to be unfertilized. When extraembryonic membranes and/or blood islands were degenerated after ~2 d of incubation, these embryos were categorized as dead embryos. The fertilized eggs with no macroscopically detectable dead embryos were considered to have had arrested within 2 d of incubation. Embryos with no heartbeat at more than 2 d of incubation were categorized as dead embryos.

### Classification of dead embryos

When embryos were observed after 18–20 d of incubation, the period of embryonic death was classified into five categories: 0–2, 2–4, 5–9, 10–11, and 12–18 d of incubation. Egg candling was performed at 4–5 d of incubation, and eggs that were not fertilized or contained dead embryos were opened at that point. The stage of embryos that died during 0-to-9 d of incubation was determined based on their morphology, and the stage of the 10–11, or 12–18 d-old dead embryos was determined based on the pigmentation pattern of skin and the development of feathers: in 10–11 d-old embryos, in which feathers were not elongated or short, increased pigments appeared mainly around the back sides of the neck, trunk, and tail. We noted that the development of hybrid embryos at 10 d of incubation seemed to be late by approximately 1 d, compared with quails, in terms of pigmentation. In the 12–18 d dead embryos, skin area with increased pigments was spread to the lateral sides of the trunk, leg, and wing, and feathers were more elongated.

### Time course analysis of the embryonic development of hybrids

The relationship between incubation time and developmental stage was investigated by staging of hybrid embryos at various incubation periods. Semen from GSP males was mainly used, and semen from Ehimejidori and BL-E males was partially used. Quail embryos and embryos of the GSP line were used as controls in this analysis. Because the number of available hybrid embryos was limited and large individual differences were found in the body weight and morphology during the late developmental stage, staging was conducted with embryos at 0 h–7 d of incubation. Stages of chickens and quails, and hybrid embryos during 0 h–7 d of incubation were classified according to the descriptions by Eyal-Giladi and Kochav (EGK)^63^ and Hamburger and Hamilton (HH)^43^,^64^. Quail embryos can be staged according to the HH and EGK chicken staging from 0 h (stage X) to 8.5 d (stage 35)^43^,^65^. Staging of embryos at given incubation periods were carried out, and the median stage at each incubation period was used for analyzing the relationship between developmental stages and incubation periods. When the median stage was two, the later stage was adopted for the analysis.

The criteria used to classify early blastoderms with abnormal morphology were as follows: blastoderms with stages similar to stage VI of chickens, during which the embryo looks like an epithelial sheet of uniform thickness, and blastoderms with stages similar to stages VII–X of chickens, during which the area pellucida is formed, were collectively regarded as stage X or earlier blastoderms. Blastoderms with stages similar to stages XI–XIV of chickens, during which the hypoblast is formed, were regarded as stage XI–XIV-like blastoderms.

To characterize the late embryonic development of hybrids, morphological observations were carried out using 17 embryos at 10 d, 5 embryos at 11 d, 3 embryos at 12 d, 2 embryos at 14 d, and 2 embryos at 16 d.

### Time-course analysis of the frequency of early developmental arrest

Hybrid embryos at 8.5–10, 12–13.5, 15, 21–36 h, and 48 h of incubation were staged according to the descriptions for EGK and HH stages. For the observation of embryos at 8.5–10 h of incubation, Ehimejidori and BL-E males were used, and GSP males were used for observations at the other incubation periods. Hybrid embryos that were morphologically similar to normal quail embryos at the same stage were classified into normal embryos. Hybrid embryos, whose morphologies were different from those of normal quail embryos at the same stage, were classified into abnormal embryos. Eleven female quails were used for the observation at 8.5–10 h of incubation, 15 for 12–13.5 h, 12 for 15 h, 6 for 21–36 h, and 7 for 48 h. Twenty-one embryos were observed for 8.5–10 h of incubation, 22 for 12–13.5 h, 40 for 15 h, 27 for 21–36 h, and 25 for 48 h.

### Molecular sexing

Molecular sexing of embryos and chicks was performed by using sequence length polymorphism of an intron of the *CHD1* gene, which is located on the Z and W chromosomes, as described elsewhere^66^. To prepare template DNA for PCR, crude DNA was obtained by a simple procedure, in which small pieces of blastoderms or early embryos were suspended in 0.2 N NaOH, incubated at 75 °C for 20 min, and neutralized by the addition of 1 M Tris-HCl pH 7.5. Alternatively, template DNA was extracted from embryos, extraembryonic tissues, or blood by a phenol-chloroform extraction method. PCR was carried out in 10-μl mixture containing 0.02 U TaKaRa EX taq (Takara Bio Inc, Otsu, Shiga, Japan), 125 μM dNTPs, 20 mM Tris-HCl pH 8.0, 100 mM KCl, 2 mM MgCl_2_, and 5 pmol of primers 2550F (5′-GTTACTGATTCGTCTACGAGA-3′) and 2718R (5′-ATTGAAATGATCCAGTGCTTG-3′). PCR was performed using a thermal cycler (GeneAmp PCR System 9700, Life Technologies, Carlsbad, CA or T100 Thermal Cycler, Bio-Rad, Hercules, CA, USA). An initial denaturing step was 94 °C for 2 min, followed by a “touch-down” scheme where the annealing temperature started from 60 °C and was lowered 1 °C per cycle until temperature reached 50 °C. The 30 cycles of the PCR reaction were performed as follows: denaturation at 94 °C for 30 s, annealing at 50 °C for 30 s, and extension at 72 °C for 30 s, and then a final extension at 72 °C for 5 min. PCR products were electrophoresed on a 2% agarose gel and stained with ethidium bromide. The 600-bp fragment derived from the Z chromosome was detected in both sexes, whereas an additional W chromosomal 450-bp fragment was amplified only in females.

### Microscopic observation and body weight measurements of embryos

Embryos were rinsed in phosphate-buffered saline (PBS) and then observed or fixed in 10% formalin in PBS. Embryos, except for those at the late stage, were photographed using a SZX7 stereomicroscope (Olympus). Large embryos at the late stage and hatched chicks were photographed using a FinePix digital camera (FUJIFILM). The weight was measured for embryos that were alive at 7–16 d of incubation and embryos that were judged to have died at 12–18 d of incubation, after fixation. The body weight of hybrid chicks was measured without fixation.

### Comparison of diameters of blastoderms at stage X between hybrids and quails

Diameters of stage X blastoderms were measured for 7 hybrids and 5 quails.

### Statistical analysis

To compare the body weight and diameter of blastoderms between hybrid males and females, a two-sided Mann–Whitney *U*-test was conducted. For multiple comparisons, Ryan’s procedure was used to calculate nominal significance levels. When the p-value was less than 0.05, the null hypothesis was rejected. To test whether the sex ratio is biased in hybrid embryos, Pearson’s chi-squared goodness of fit test was used, and the alpha level was 0.05. To test whether frequencies of abnormal blastoderms at 15 h incubation differ between the sexes and whether malformation frequencies differ between the sexes, Fisher’s exact tests were conducted using a two-sided alpha level of 0.05. Hybrids with unknown sex were ignored in the chi-squared and Fisher’s exact tests.

## Additional Information

**How to cite this article**: Ishishita, S. *et al*. Embryonic development and inviability phenotype of chicken-Japanese quail F_1_ hybrids. *Sci. Rep.* **6**, 26369; doi: 10.1038/srep26369 (2016).

## Supplementary Material

Supplementary Information

## Figures and Tables

**Table 1:** Developmental status of hybrids between quails and chickens (the GSP line of Fayoumi) at various incubation times.

**Crosses**	**Quails**^1^	**Fertile quails**^2^	**Egg collection**^3^ **(days)**	**AI**^4^ **(times)**	**Incubated eggs**^5^	**Fertilized eggs**^6^	**Stages of developmental arrest**^7^						**Hatched**^8^
							**Membrane**	**Membrane & blood**	**2‒4 d**	**5‒9 d**	**10‒11 d**	**12‒18 d**	
Quail × Chicken	29	27	78	27	1664	405	39	262	20	23	5	39	17
			Frequency for incubated eggs			24.3%	2.3%	15.7%	1.2%	1.4%	0.3%	2.3%	1.0%
			Frequency for fertilized eggs			–	9.6%	64.7%	4.9%	5.7%	1.2%	9.6%	4.2%
								Male	2	6	3	19	17
								Female	3	17	2	20	0
								n.d.	15	0	0	0	0

Quail eggs artificially inseminated with chicken semen were incubated for 18‒20 d, and fertilization and embryonic development were examined (see Methods). Incubation times at which embryonic development was arrested were estimated from morphologies of dead embryos (see Methods).

Male, number of males; Female, number of females; n.d., not determined.

^1^Number of female quails used for experiments.

^2^Number of female quails that laid fertilized eggs.

^3^Days of egg collection.

^4^Number of artificial inseminations.

^5^Number of incubated eggs.

^6^Number of fertilized eggs.

^7^Number of fertilized eggs that showed developmental arrest. Membrane, developmental arrest with extraembryonic membrane and no embryos; membrane and blood, developmental arrest with extraembryonic membrane, blood island, and no embryos; 2‒4 d, embryonic death during 2‒4 d; 5‒9 d, embryonic death during 5‒9 d; 10‒11 d, embryonic death during 10‒11 d; 12‒18 d, embryonic death during 12‒18 d.

^8^Number of newborn chicks.

**Table 2:** Developmental status of quails at various incubation times.

**Crosses**	**Quails**^1^	**Fertile quails**^2^	**Egg collection**^3^ **(days)**	**AI**^4^ **(times)**	**Incubated eggs**^5^	**Fertilized eggs**^6^	**Stages of developmental arrest**^7^						**Hatched**^8^
							**Membrane**	**Membrane & blood**	**2‒4 d**	**5‒9 d**	**10‒11 d**	**12‒18 d**	
Quail × Quail	3	3	45	–	96	70	0	4	0	1	0	7	58
			Frequency for incubated eggs			72.9%	0%	4.2%	0%	1.0%	0%	7.3%	60.4%
			Frequency for fertilized eggs			–	0%	5.7%	0%	1.4%	0%	10.0%	82.9%
								Male	0	0	0	0	26
								Female	0	1	0	6	32
								n.d.	0	0	0	1	0

Quail eggs were obtained from female quails that were mated naturally with male quails, and were incubated for 17–20 days. See Table 1 legend for explanation of column headings.

Male, number of males; Female, number of females; n.d., not determined.

**Table 3:** Sex ratio of hybrid embryos at various incubation times.

**Incubation time**	**Males**		**Females**		**Male frequency**	p-value*	**χ^*2*^**
	**Observed**	**Expected**	**Observed**	**Expected**			
2 d	47	44.5	42	44.5	52.8%	0.596	0.281
5 d	45	42	39	42	53.6%	0.513	0.429
10 d**	39	30.5	22	30.5	63.9%	0.030	4.738
12 d**	36	28	20	28	64.3%	0.033	4.571
Newborn chicks	17	8.5	0	8.5	100.0%	n.e.	n.e.

The GSP line of Fayoumi was used for artificial insemination.

The numbers of hybrid males and females that were alive at 2, 5, 10, and 12 d were estimated, based on the numbers of hybrid embryos that were judged to have died at 2‒4, 5‒9, 10‒11, and 12‒18 d and were hatched. Fifteen embryos at 2 d of incubation, with unknown sex, were not used for statistical analysis.

n.e., not examined

^*^Pearson’s chi-squared goodness of fit test.

^**^Significant difference between the observed and expected frequencies for *P* < 0.05.
