# Supplementary material for: Embryonic development and inviability phenotype of chicken-Japanese quail F1 hybrids
Source: Sci Rep. 2016 May 20;6:26369. doi: 10.1038/srep26369 (PMC4873824; doi:10.1038/srep26369)
Supplement: Supplementary Information [file srep26369-s1.pdf]

Supplementary Information

Embryonic development and inviability phenotype of chicken-Japanese quail F_1 hybrids

Satoshi Ishishita[1], Keiji Kinoshita[1], Mikiharu Nakano[1], Yoichi Matsuda[1,2*]

[1]Avian Bioscience Research Center, Graduate School of Bioagricultural Sciences,

[2]Laboratory of Animal Genetics, Department of Applied Molecular Biosciences, Graduate School of Bioagricultural Sciences, Nagoya University, Furo-cho, Chikusa-ku, Nagoya, Aichi, 464-8601, Japan

* Corresponding author.

Table S1. Developmental status of hybrid embryos between Japanese quails and chickens (Ehimejidori and NH-413) at 3 d of incubation

Trial	Chicken strain/breed	Quails[1]	Fertile quails[2]	Egg collection[3] (days)	AI[4] (times)	Incubated eggs[5]	Fertilized egg[6]	Membrane[7]	Membrane & Blood[8]	Dead embryo[9]	Living embryo[10]
1	Ehimejidori	7	7	31	7	191	58	11	25	7	15
	NH-413	8	7	31	7	209	63	16	25	15	7
2	Ehimejidori	38	37	76	24	1944	611	291	208	20	92
	Total	-	-	-	-	2344	732	318	258	42	114
				Frequency for incubated eggs			31.2%	13.6%	11.0%	1.8%	4.9%
				Frequency for fertilized eggs			-	43.4%	35.2%	5.7%	15.6%

Quail eggs artificially inseminated with chicken semen were incubated for 3, 5, or 7 d, and fertilization and embryonic development were examined (see Methods). Stages at which embryonic development was arrested were estimated from the morphology of dead embryos (see Methods).

[1]Number of quail females used for artificial insemination.

[2]Number of quail females that laid fertilized eggs.

[3]Days of egg collection during each trial.

[4]Number of artificial insemination during each trial.

[5]Number of incubated eggs.

[6]Number of fertilized eggs.

[7]Number of fertilized eggs that showed developmental arrest with extraembryonic membrane and no discernible embryos.

[8]Number of fertilized eggs that showed developmental arrest with extraembryonic membrane, blood island, and no discernible embyos.

[9]Number of fertilized eggs with discernible embryos that were dead at 3 d of incubation.

[10]Number of fertilized eggs with discernible embryos that were alive at 3 d of incubation.

Table S2. Developmental stages of chickens, quails, and their hybrids

Hours of incubation	Chicken	Quail	Hybrid
7.5–8	-	-	St. XI (1)<br>St. XII (2)<br>St. XIII (3)<br>St. XIV (1)
12	St. 3 (2)	St. 2 (1)<br>St. 3 (4)	XIII (4)<br>XIV (1)<br>2 (3)
24	St. 5 (4)	St. 6 (4)	St. 4 (2)
48	St. 12 (1)<br>St. 13 (1)	St. 13 (3)	St. 9 (1)<br>St. 10 (2)<br>St. 11 (1)<br>St. 13 (2)
72	St. 16 (1)<br>St. 17 (1)<br>St. 18 (3)	St. 16 (1)<br>St. 17 (1)<br>St. 18 (2)<br>St. 19 (6)	St. 15 (1)<br>St. 16 (5)<br>St. 17 (8)
96	St. 24 (2)	St. 24 (6)	St. 19 (2)<br>St. 20 (1)
120	St. 27 (4)	St. 27 (3)	St. 23 (2)<br>St. 24 (1)<br>St. 25 (1)
144	St. 29 (3)	St. 29 (1)	St. 27 (2)
168	St. 31 (4)	St. 33 (6)	St. 29 (5)<br>St. 30 (1)
Hatching	Day 21 (5)	Day 17 (8)<br>Day 18 (19)<br>Day 19 (1)	Day 18 (6)<br>Day 19 (3)<br>Day 20 (6)<br>Day 21 (2)

For staging of chicken and quail embryos, GSP line chickens and commercial quails were used. Hybrid embryos (GSP males × commercial quail females) were used for all observations, except for those at 7.5–8 h of incubation (Ehimejidori or BL-E males × commercial quail females) and 168 h of incubation (Ehimejidori males × commercial quail females). The number of hybrid embryos is indicated within parentheses.

Table S3. Sex ratio of living hybrid embryos at various incubation times

Incubation time	Paternal strain	Males		Females		Male frequency	p-value*	χ^2
		Observed	Expected	Observed	Expected			
0 h	GSP	25	28.5	32	28.5	43.9%	0.354	0.860
2–3 d	NH-413 or Ehimejidori	16	13.5	11	13.5	59.3%	0.336	0.926
5 d	Ehimejidori	15	13	11	13	57.7%	0.433	0.615
7 d	BL-E or Ehimejidori	24	20.5	17	20.5	58.5%	0.274	1.200

One embryo at 5 d of incubation, with unknown sex, was not used for chi-squared test.
*Pearson's chi-squared goodness of fit test.

Table S4. Sex ratio of malformed hybrid embryos

Sex	Number of embryos at 2–4 d or more			Number of embryos at 10 d or more		
	Normal	Malformed	Total	Normal	Malformed	Total
Male	37	10	47	30	9	39
Female	26	16	42	14	8	22
n.d.	15	0	15	0	0	0
Total	78	26	104	44	17	61

n.d., not determined.

Table S5. Summary of artificial insemination

Sampling periods				Egg collection[1] (Days)	AI[2] (times)	Chicken breed/line[3]	Quail group[4]	Number of quails[5]	Number of fertile quails[6]	Number of incubated eggs[7]	Number of fertilezed eggs[8]	Examination items
1	3/31/2010	–	4/30/2010	31	7	EJ	A	15	14	400	121	Fertility and survivability at 3 d. Sex ratio of 2–3-d living embryos.
2	7/23/2010	–	11/24/2010	76	24	EJ / NH-413	B	38	37	1944	611	Fertility and surviability at 3 d. Sex ratio of 5-d living embryos.
3	7/19/2012	–	2/25/2013	81	48	GSP	C	9	9	401	213	Developmental stage. Early embryogenesis at 21–36 h. Sex ratio of 0-h blastoderms.
4	9/1/2012	–	4/8/2013	112	56	GSP	D	24	19	333	254	Developmental stage. Morphology and body weight of 12-d, 14-d, and 16-d living embryos.
5	10/7/2012	–	4/13/2013	75	45	GSP	E	12	9	574	200	Developmental stage. Morphology of 12-d living embryos.
6	6/3/2013	–	10/8/2013	106	21	BL-E	F	12	10	594	168	Sex ratio of 7-d living embryos.
7	7/18/2013	–	10/8/2013	60	10	BL-E	G	12	7	239	41	Sex ratio of 7-d living embryos. Early embryogenesis at 7.5–10 h.
8	8/25/2013	–	12/29/2013	34	5	BL-E	H	8	8	203	38	Sex ratio of 7-d living embryos. Early embryogenesis at 7.5–10 h.
9	10/24/2013	–	12/8/2013	27	12	EJ	I	15	11	232	52	Sex ratio and body weight of 7-d living embryos.
10	2/4/2014	–	3/14/2014	37	15	EJ	H	2	1	11	7	Body weight of 10-d living embryos.
11	2/4/2014	–	5/1/2014	68	27	EJ	J	16	11	261	51	Morphology and body weight of 10-d living embryos.
12	5/10/2014	–	5/30/2014	13	5	EJ	J	3	1	5	2	Developmental stage. Sex ratio and body weight of 7-d living embryos.
				13	5	EJ	K	16	13	155	31	
13	6/1/2014	–	10/13/2014	78	27	GSP	J	2	2	113	27	Fertility, surviability at various incubation periods, and hatchability. Temporal changes of sex ratio. Frequency of malformed embryos. Body weight of 12–18-d dead embryos and hatched hybrids.
				78	27	GSP	K	28	26	1556	382	
14	6/17/2015	–	7/15/2015	24	6	GSP	L	9	8	59	38	Developmental stage. Early embryogenesis at 12–15 h. Sex ratio of stage XI–XIV-like blastoderms.
15	6/24/2015	–	7/15/2015	17	4	GSP	M	14	7	47	22	Developmental stage. Early embryogenesis at 12–15 h. Sex ratio of stage XI–XIV-like blastoderms.
16	8/19/2015	–	8/27/2015	9	3	GSP	M	7	7	61	25	Developmental stage. Early embryogenesis at 48 h.
Total				939	347			242	200	7188	2283	

[1]Days of egg collection.
[2]Number of artificial insemination (AI).
[3]Chicken breed or line used for AI.
[4]Names of female quail groups used for AI.
[5]Number of female quails used for AI.
[6]Number of female quails that laid fertilized eggs.
[7]Number of incubated eggs.
[8]Number of fertilized eggs.
EJ, Ehimejidori.

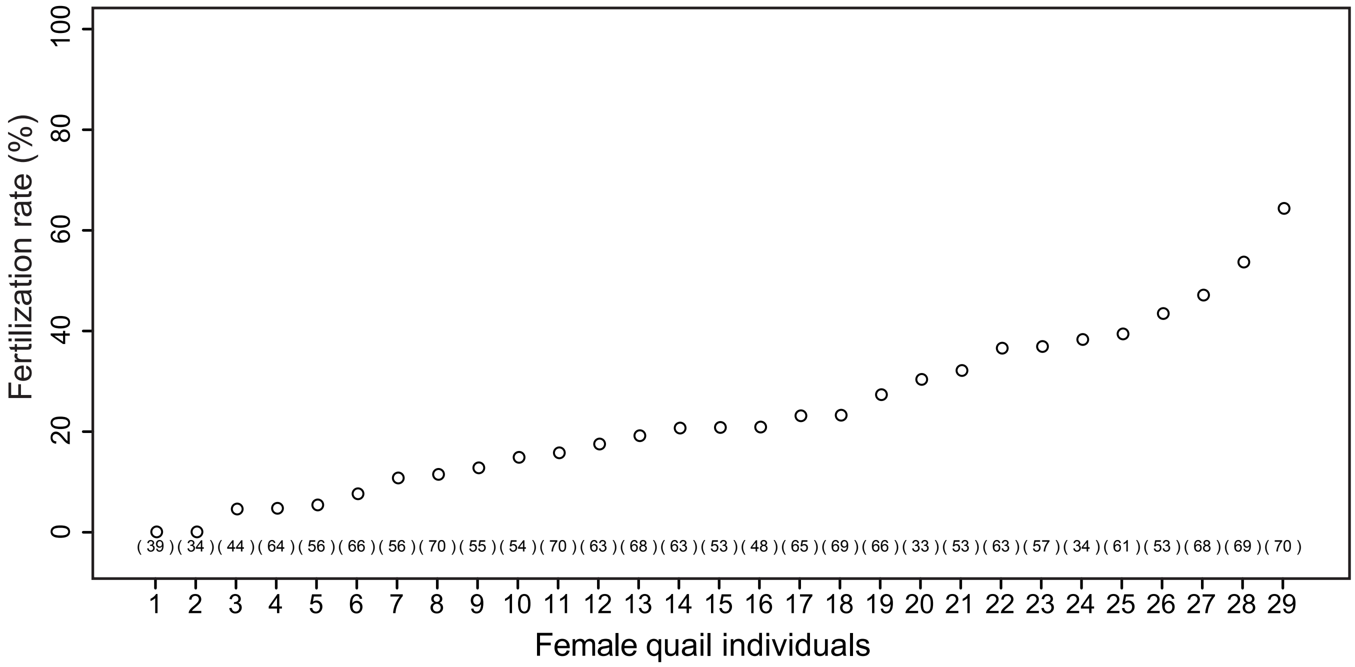

Figure S1. Fertilization rates in interspecific crosses between female quails and male chickens. The horizontal axis indicates individual identification numbers of female quails. Each point represents a percentage of fertilized eggs in incubated eggs. The number within each parenthesis is the number of incubated eggs.

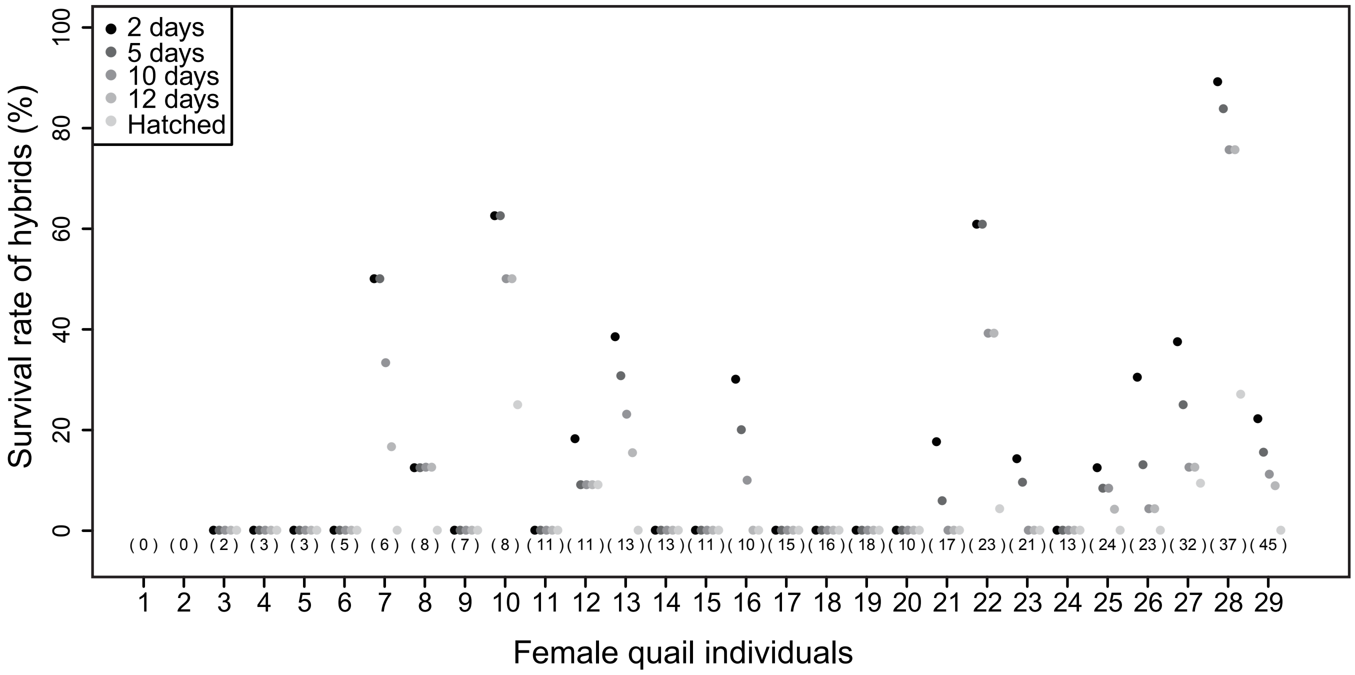

Figure S2. Survival and hatching rates of interspecific hybrids. The horizontal axis indicates individual identification numbers of female quails. Each point represents a percentage of living embryos at 2, 5, 10, or 12 d of incubation or hatched hybrids in fertilized eggs. The number within each parenthesis is the number of fertilized eggs.

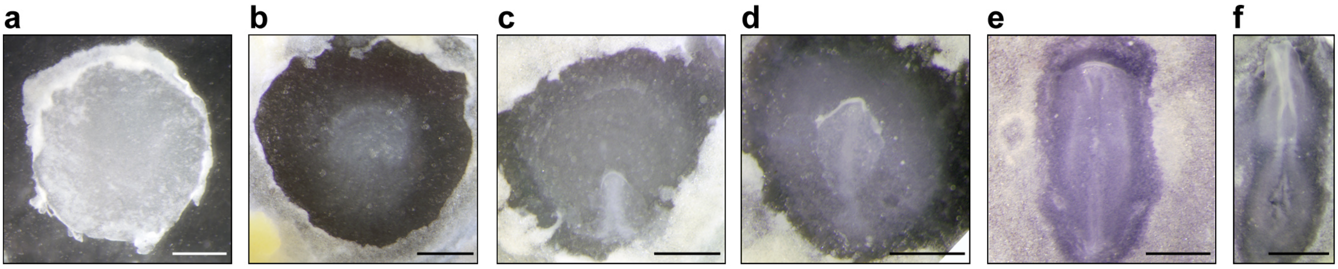

Figure S3. The development of quail embryos during the early stage. a–f, Representative images of quail embryos of stage X at 0 h (**a**), stage XIII at 9 h (**b**), stage 2 at 9.5 h (**c**), stage 3 at 18 h (**d**), stage 6 at 24 h (**e**), and stage 8 at 27 h (**f**) of incubation. Scale bars, 1 mm.

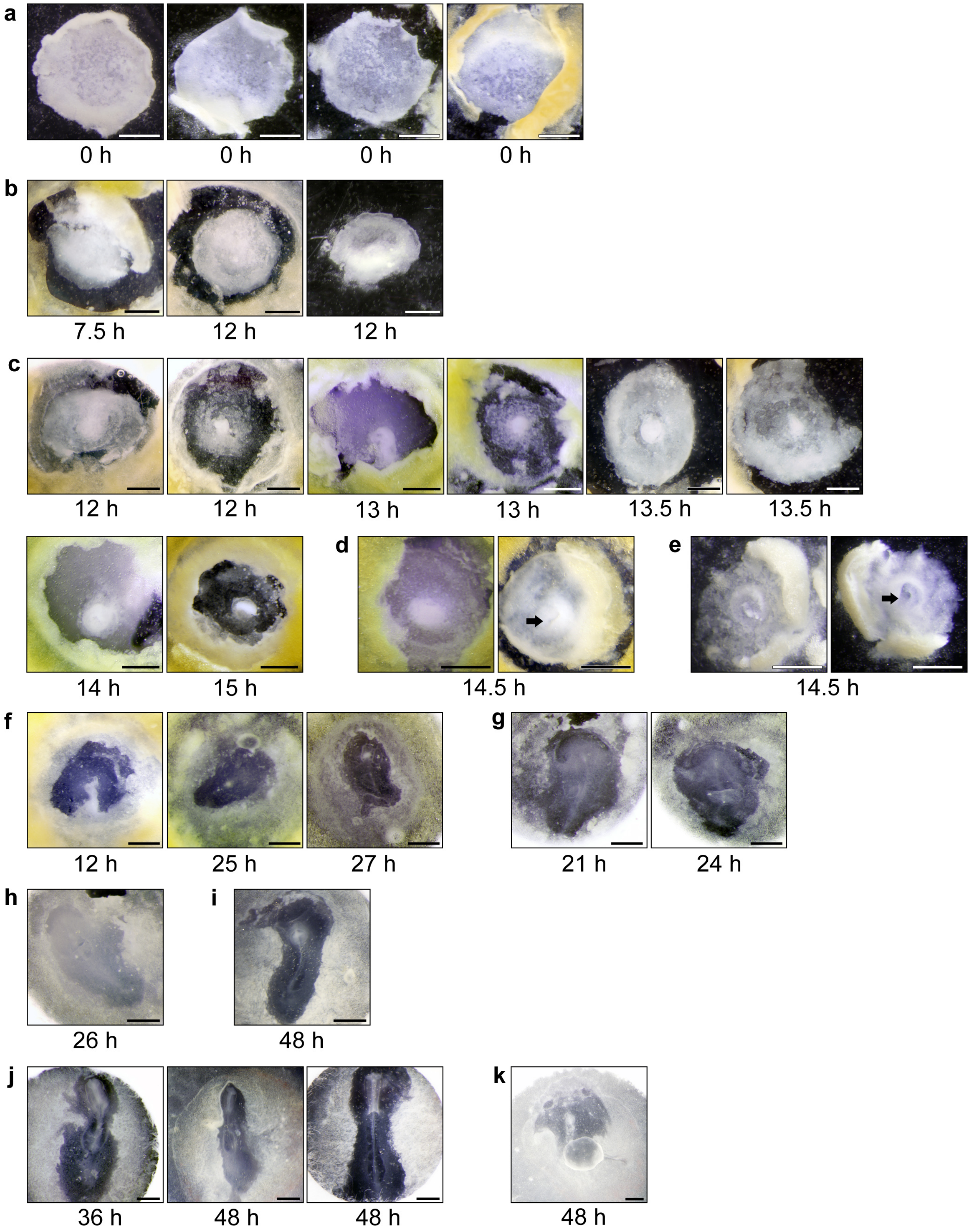

Figure S4. Representative images of hybrid embryos at 0–48 h of incubation. **a**, Stage X blastoderms with normal morphology. **b**, Blastoderms at stage X or earlier with abnormal morphology. **c**, Stage XI–XIV-like blastoderms with abnormal morphology. **d**, **e**, Ventral (left panels) and dorsal (right panels) views of stage X–XIV-like abnormal blastoderms. The central regions of the ventral sides of area pellucida are swollen and cavities (arrows) can be observed. **f**, Abnormal blastoderms at stages similar to 2 (left), 3 (middle), and 4 (right). **g**, The body axes of the stage 4-like blastoderms are not straight. **h**, **i**, Stage 5–6-like blastoderms with normal morphology (**h**) and with abnormal morphology (**i**). **j**, Abnormal embryos with stages equivalent to 9 (left and middle) and 10 (right). **k**, Degenerated embryo whose stage could not be determined. The incubation period is shown below each panel. All embryos except for those shown in the right panels in (**d**) and (**e**), and in the left and middle panels in (**j**), were photographed ventral side up. Scale bars, 1 mm.

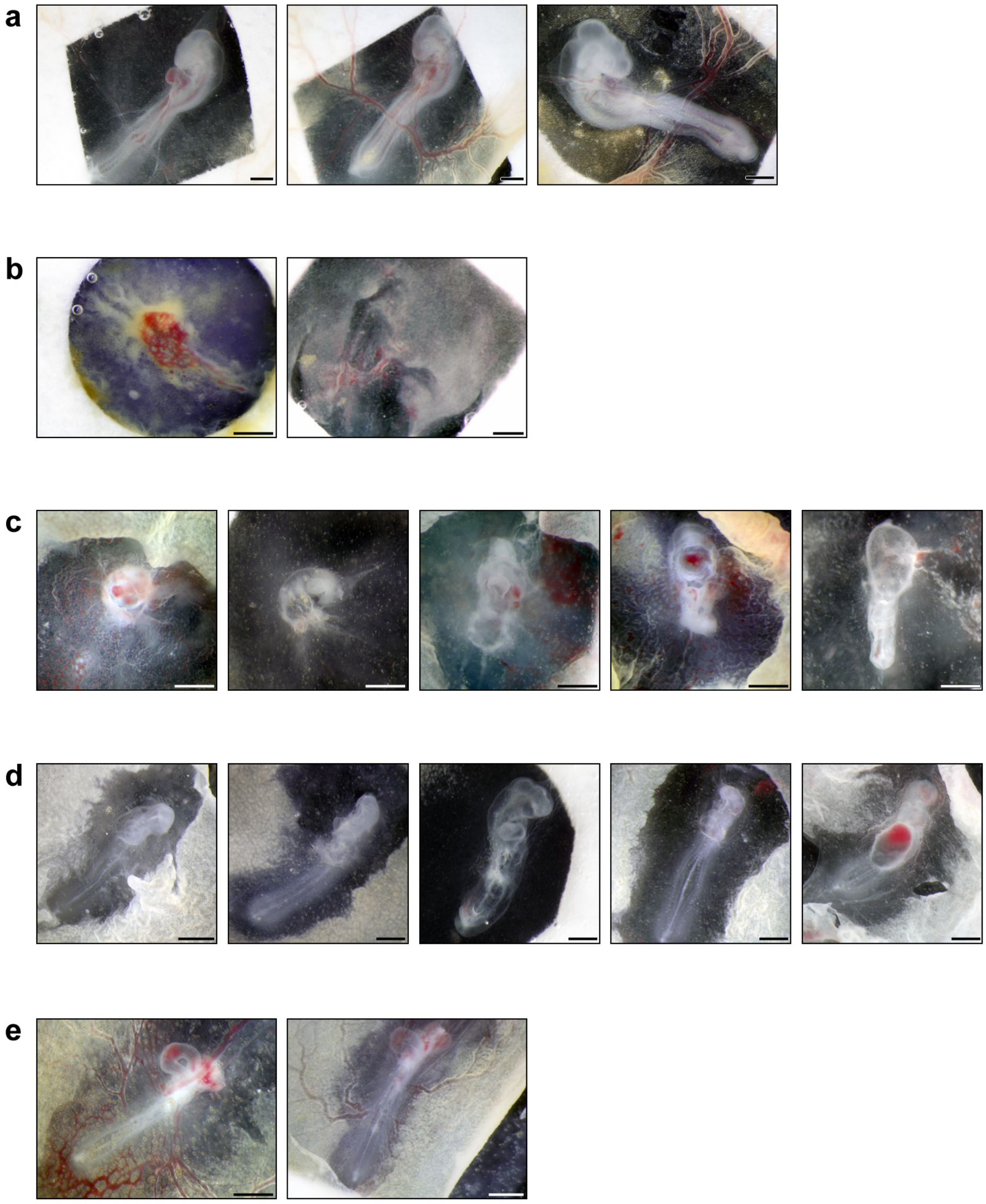

Figure S5. Representative images of hybrid embryos at 72 h of incubation. a, Embryos with normal morphology. **b**, Degenerated blood vessels and no discernible embryonic structures. **c**, Degenerated embryos whose stages could not be determined. **d**, Degenerated embryos with stages equivalent to the somite-to-pre-circulation stage. **e**, Post-circulation stage embryos exhibiting abnormal head development. All embryos except for those shown in (**c**) were photographed ventral side up. It is uncertain whether the images shown in (**c**) were taken from the dorsal or ventral side of the embryos. Scale bars, 1 mm.

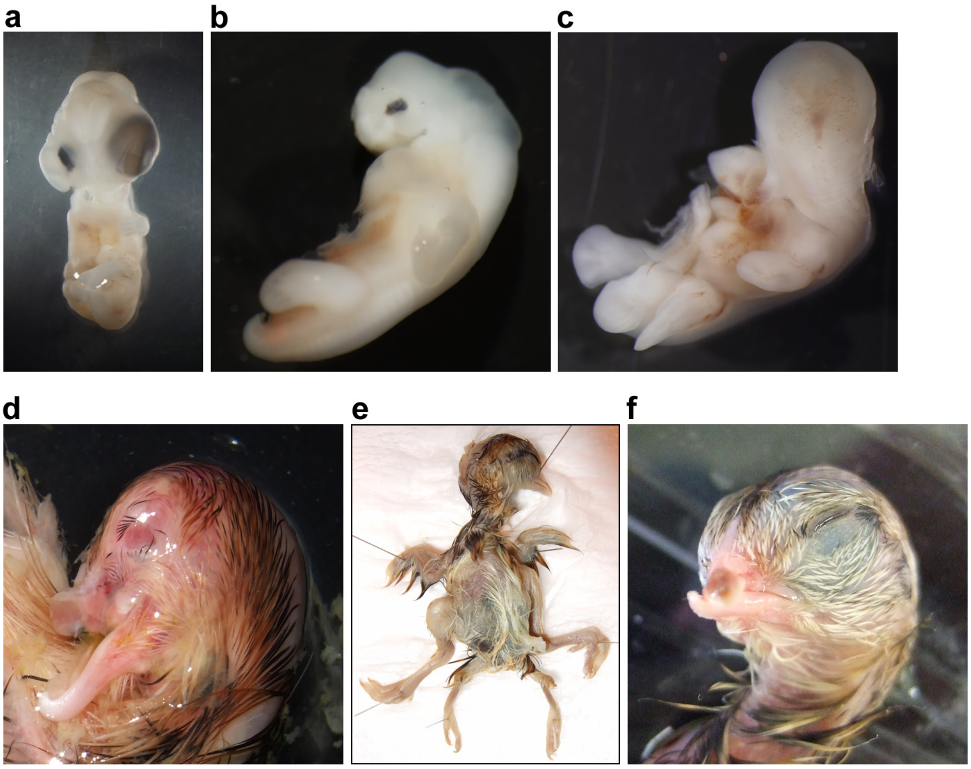

Figure S6. Malformations in hybrid embryos. a–c, Representative images of hybrid embryos at 7 d of incubation, exhibiting abnormal eye development (**a**), abnormal head development (**b**), and general malformation (**c**). **d**, **e**, Dead hybrid embryos at 12–18 d of incubation, exhibiting abnormal head development (**d**) and an additional pair of hind limbs (**e**). **f**, Representative image of malocclusion in hybrids that died prior to hatching.
